# Social configurations in the moment of post-foundationalism

**DOI:** 10.3389/fsoc.2022.1078011

**Published:** 2023-01-26

**Authors:** Abbas Jong

**Affiliations:** Department of Social Sciences, Humboldt University of Berlin, Berlin, Germany

**Keywords:** social configuration, post-foundationalism, contingency, Norbert Elias, historical constellation, orders of category

## Abstract

Modern social sciences arose during a period of classical modernity in which discovering universal rules between distinct phenomena was the most prominent criterion of scientific knowledge. Social phenomena were considered in the form of isolated, determined, standardized, and regulated objects whose knowledge, like that of the natural sciences, depended on the understanding of universal laws. The accidental and the contingent were eliminated in favor of universal laws. With the intensifying of modernity and the transition to late and liquid modernity, and by suspending many dominant cognitive categories, this kind of essentialist foundationalism was attacked by a variety of anti/non-foundationalist criticism that subscribed to either plural grounds or groundlessness, a bottomless ground in which scientific knowledge at a high level lost its significance. This predicament has given rise to several biases and antinomies in modern social theory. By addressing some of these predicaments and antinomies, including foundationalism/non(anti-)foundationalism, agency/structure, the individual/society, essentialism/relativism, and universalism/singularism, the present article strives to propose the idea of social configurations as a solution to overcome them, and through this endeavor, it is indicated that considering these configurations can effectively explain emerging and interrelated global phenomena. By prioritizing the conditions of possibility for social phenomena, and taking into account their contingency, as well as the incompleteness and partiality of their foundations, social configurations are considered as units at the level of the particular whose relationality, indeterminacy, interdependence, and fluidity constitute their central features.

## 1. Introduction

Social science emerged in a historical as well as a cognitive context in which the natural sciences were dominant at a high level (Wallerstein, 1996; Mahoney, 2021). According to the ontological foundations of these sciences (mainly based on the Cartesian point of view), there was an independent reality beyond and outside of the subject. In addition to independence, out-thereness, and anteriority, these realities were considered as determined entities, and therefore, a set of definite forms and relations were assumed for them. The world of these realities was common, shared, universal, stable, and the same everywhere, and therefore, the knowledge of their universal and valid laws—the validity given by the sciences themselves—was the most important criterion for being scientific knowledge (Law, 2004, p. 23–26). But this kind of confrontation with reality has implications for their creation. The presupposition of order, stability, and regularity for social realities, as well as their consideration in the form of definite units, both evoked and created a particular aspect of the social world. At another level, grasping the order and regulation of realities allows the modern subject to change this order according to his/her will. More specifically, and according to the characteristics of the modern episteme (Foucault, 1966), which provided the conditions of the possibility for the modern social and human sciences, a kind of ground or essence, as well as origin, was conceived for divided and isolated social phenomena. The key to comprehending the regulated and standardized phenomena, for which history might be considered, was to identify this foundation. It was in these cognitive coordinates that categories such as society, culture, nation, government, class, citizen, etc. either constructed or found new implications. By reifying and presupposing these categories, the initial mainstream social sciences attempted to place multiple and indeterminate concrete phenomena under the categories, and draw on formal rationality, to make them comprehensive. This effort, in addition to highlighting some aspects of the realities, actively participates in their creation. All of this took place in a context in which modern immanent reason claimed to understand and construct a new rational order.

But developments in the ontological status of contemporary society and the institutionalization of a kind of widespread uncertainty throughout the world (Jong, 2022b) have caused the dominant social categories to lose their explanatory significance. The acceleration and integration of social forces and elements at the global level have reached such a point that it is almost impossible to define the social based on the previous foundations and settings. If previously the social, as a unit, or a set or a domain, could be easily closed and its elements could be identified, now the constituent elements of the social have become so numerous and indeterminate to the extent that no closed point or boundary can be imagined. In particular, this is clearly evident in transnational and cosmopolitan phenomena (Jong, 2022a). With the dominance of complexity, mobility, liquidity, and diversity as prominent parameters of the new global social terrain, the contextual conditions of constructing the social, its basic constituents as well as the internal relations of different parts of social phenomena, their speed and forms of transformation have veered dramatically. Novel social realities and dynamics are permanently crossing, reconstructing, transcending, and transforming existing social boundaries, units and categories, whether they are localities, regions, nations, trans-nations, empires, or are partial to the world system (Khagram and Levitt, 2007; Knott and McLoughlin, 2010; Jong, 2016a).

Grasping and understanding the novel social terrain as well as new phenomena, forces, dynamism, and elements requires new categorization and conceptualization apparatus that can be sensitive to the specificities and uniqueness of the phenomena, of which indeterminacy, fluidity, and endlessness are the main operative features (Albrow, 1996; Bauman, 2000a; Shaw, 2000; Beck, 2002, 2016a,b; Beck and Sznaider, 2006; Vertovec, 2009, 2015; Rehbein, 2015; Reckwitz, 2020a,b). It seems that the metamorphosis of the world reveals itself more than ever in different layers of the social world, but maybe it is the social sciences that have been immune from this process (Beck, 2016c). Global environmental crises, emerging mass revolutions and rebellions, the emergence of new forms of transnational constructions and forces, emerging crises for established nation-states, fluid realities generated by the global growth of technology, the increase of global mass migrations and inequalities, the globalization of risks, the unbridled expansion of neoliberalism, new forms of autocracy, etc., and in general, the emerging global configuration cannot be easily understood and explained by existing social sciences. The area that environmental crises cover is neither a nation-state nor a local society but a vast geographical territory with several social actors and unexpected consequences. But the social sciences and their traditional categories have nothing to offer, neither in terms of knowledge nor in terms of policy recommendations. The new mass protests and uprisings around the world, which are mainly formed in the light of communication technology and social networks, are still explained and interpreted in the social sciences under different theories of social movements. The theories, categories, and central notions of the social sciences are disproportionate and primitive for a changing and highly interconnected world. In an analytic language, existing social categories, such as citizen, race, class, gender, ethnicity, nationality, and globality; units of analysis, such as actors, family, household, nation, system, state, and structure and scales of analysis, such as local, national, regional, transnational, global, and cosmopolitan, at a high level, are losing their significance in explaining the emerging phenomena. We are facing multifaceted, ongoing entities which fall into one or two categories, but at the same time and from different aspects, suspend those categories and go beyond them. A phenomenon is constructed at the national level, but it has consequences at the global level, and on the contrary, a global phenomenon could accompany multiple implications at the local and national levels. A phenomenon at one level and at a particular period of time has cultural implications, but at another level and another period crystallizes itself as an economic reality. A government may be authoritarian in some respects, but democratic in others.

By identifying similarities or differences, either in a deductive or inductive mode or by finding a given ground, what conventional social theory has done so far-apart from some pseudo-philosophical efforts of classical, neo-Kantian sociological theorists such as Max Weber, George Simmel, and then contemporary phenomenological theorists like Peter Berger-has been to categorize multiple and distinct phenomena into universal, solid and given categories (Akiwowo, 1988; Long and Fox, 1995; Strauss, 2017). What is assumed in most of these theories is that the social world, like the natural one, consists of entities that have internal essences, “which endow the entities with an identity and a certain nature” (Mahoney, 2021; p. 1). For social scientists, social objects are equivalent to separate, standardized, self-contained, and regulated-based entities (Elias, 1984; Bauman, 2002; Luhmann, 2012; Reckwitz, 2020b), and they treat their analytical categories, in another aspect, “as corresponding to things ‘out there' in the external world that possess properties and dispositions” (Mahoney, 2021; p. 1; Lyotard, 1986). By highlighting the singular and multiple characters, on the contrary, many “post” (modern)—anti-essentialist—theories and perspectives in social sciences have attempted to either pluralize the categories or in general, denied the existence of universal social categories (Lyotard, 1986; Fairlamb, 1994; Sayer, 1997; Baudrillard, 2007), approaches whose translations at the level of empirical research lead to a situation in which, as Ulrich Beck put it, “sociology falls into the trap of presentism” (Beck, 2016c; p. 147). Proceeding on this basis, it could be argued that the dominant social inquiry is framed around a fundamental distinction, “the distinction between theory—‘which equates theory with universalistic theory'—and diagnosis of the present age” that is “time-blind”, “context-blind” and indifferent to transformations (Beck, 2016c; p. 148).

In this respect, without falling into the trap of essentialism as well as extreme relativism and presentism, touching on social phenomena requires revisiting some epistemological premises in social science in order to make sense of the indeterminacy, contingency, and endlessness of the emerging phenomena. Put differently, how can we approach social phenomena in a post-foundational society, a society where all that is solid melts into the air? (Berman, 1988). Additionally, in Beck's words, “what happens when the premises and boundaries that define the units of empirical research and theory disintegrate?” (Beck and Sznaider, 2006; p. 393). Any attempt to deal with this issue has to begin its analysis by scrutinizing and criticizing a number of antinomies, predicaments, or biases that have largely dominated the axioms of modern social sciences and theories. Through inquiring into five antinomies and biases, namely foundationalism as opposed to anti/none-foundationalism; the individual contrary to society; agency vs. structure; essentialism vs. relativism; and universalism contrasted with singularism, the current article aims to address this issue and will argue for third positions in going beyond them. Although all these antinomies and predicaments can be examined in relation to the predicament of foundationalism and anti/non-foundationalism, they are closely interrelated to each other and mutually strengthen one another in different ways. Thematically, this article consists of three coherent parts. In the first section, it presents an overview of the current state of the social sciences, producing a diagnosis of a contemporary “post-foundationalist” moment, in which many lines of traditional social theoretical inquiry are taken to have been de-legitimated because of the various antinomies they find themselves in. By introducing Norbert Elias's figurational approach as a possible route out of those antinomies and drawing on the work of Boike Rehbein as an Adorno-, Foucault-influenced corrective to some problems of figurational theory, it then proposes the idea of social configuration. In the third section, some of the implications of this post-foundationalist framework are explored with reference to various objects of social inquiry.

The rest of this article is structured as follows: First, foundationalism and its crisis in social theory as well as some reactions to the crisis will be addressed. Drawing on the notion of post-foundationalism, which is formulated by Marchart (2007), the article explains how the notion, by shifting attention from social phenomenon as the final, major object of social inquiry to the exploration of the conditions of (im)possibility of the object, is striving to put forth a solution for the antinomy of foundationalism and anti/non-foundationalism. Second, it will be shown how the previous predicament manifests itself through the antinomies of the individual/society or agency/structure and universalism/singularism. Drawing on Norbert Elias' notion of “figuration,” and by addressing the Idea of “kaleidoscopic dialectics” which is proposed by Rehbein (2015, 2018), the article suggests that, by considering social reality as a social configuration in a particular level, many issues, biases, and antinomies, which are addressed in previous parts, can be suspended in the favor of a position in which contingency, indeterminacy, and the relationality of the realities are the main pillars. Then, the article proceeds to take into account the predicament of essentialism/relativism. By referring to the Bourdieusian relational approach (Bourdieu, 1990a,b; Bourdieu and Wacquant, 1992; Lamont and Molnár, 2002; Wimmer, 2002; Brubaker, 2004; Todd, 2005; Mohr, 2013) in addressing the construction of social realities, the procedural and contingent quality of the realities takes precedence. In these circumstances, social realities, instead of being essential or impossible, are considered possibilities that are constructed in a particular moment and condition, based on a network of intertwined and uneven relations. The realities are contingent as long as the conditions of their possibility and their relationships change, thus, they are never established or completed. Finally, I conclude with some additional thoughts on what the “post-foundationalist approach” brings to understanding the contemporary social world.

## 2. Contingency as the condition of (im-) possibility of the social

From the advent of sociology in particular and modern social theory in general, as Bauman (2002) put it, society was portrayed by sociologists as an “imaginary entity,” but this entity was imagined “at the time of ‘solid modernity', of building tough frames and enclosures meant to last, of integration and unification” (Bauman, 2002; p. 43). According to him, “it was the time of welding archipelagoes of scattered communal islands into the compact continents of nation-states, of cementing diffuse and variegated dialects, customs and ways of life into one nation of one language, purpose, and government” (Bauman, 2002; p. 44). Although at the time of its formation, society may have consisted of the interactions of individuals in the realm of everyday life, at another level, it transcended the individuals and constructed collective structures. Here, society was imagined as a self-enclosed, determinate, and trans-individual entity, the entity whose perceived boundaries and solidarity were to be guaranteed by the socialization of individuals as well as various regimes of social control. According to Bauman, sociology, as a modern phenomenon, “set about eliminating the accidental and the contingent” (Bauman, 2002; p. 28). “If the notorious ‘project of modernity' can be adumbrated at all,” as he put it, “it can only be envisaged as a retrospective gloss on a firm intention to insert determination in the place where accidents and games of chance would otherwise rule” (Bauman, 2002; p. 28). In modern social theories, society is presupposed as a self-immanent, isolated entity that is prone to equilibrium, emphasizing the means by which society ensures its existence and controls the distributing actors. Therefore, through different directions, independence and stability reflected as a natural and prior position ended up reducing the procedural and relational traits of social phenomena to static, universal, and fixed categories (Elias, 1984; Luhmann, 2012).

What was common among social theorists at the high level was their perception of society and the social as definite, self-contained, and determined (objective or subjective) objects. When Emile Durkheim wrote that “the determining cause of a social fact should be sought among antecedent social facts and not among the states of individual consciousness” (Durkheim, 1982; p. 134), for instance, it refers to the very proposition that society and the social were both source and purpose of social explanation, that is, the social theorists either referred to the social to understand other phenomena or tried to understand the social in terms of another social. The same logic could be found among other social theories, such as economics, behaviorism, positivism, and structuralism. Marchart (2007) employs the term foundationalism to name those social theories in which a principle is sought which is to ground social explanation from without. It is from this transcendent ground that the function of the social, as well as other phenomena, is claimed to be derived (Marchart, 2007; p. 11-12). In general, those social theories which imply that different social phenomena are “grounded on principles that are (1) undeniable and immune to revision and (2) placed outside and beyond the phenomena,” can be classified as foundationalism (Herzog, 1985; p. 20; Marchart, 2007; p. 11). By assuming the presence of a permanent and stable foundation, here, understanding of social phenomena becomes conditional and also reduced to a quest for a fixed, ground zero base (Strauss, 2017). As an epistemological issue in philosophy, foundationalism has emerged through a philosophical controversy over “the question of whether or not knowledge and truth must have a foundation” (Sosa, 1980; p. 547). In justification of knowledge, foundationalists believe in a solid foundation and are looking for it on rational foundations or factual evidence (Sosa, 1980, 1998). In Marxism and non-Marxism economic determinism, for instance, first, “a set of principles” (here the economic laws) will be prepared, principles which are envisaged as the essence and nature of culture or politics (or what politics or culture really are) and, then, this “ground,” namely the economic base, is located outside of, or beyond, the realms of politics and culture. Hence, the realms of culture and politics are reduced to a “merely super-structural” affair (Marchart, 2007; p. 12).

By rejecting absolute certainty for knowledge and existing trans-historical and solid ground, many non/anti-foundationalists assert that it can be impossible to “provide knowledge with secure foundations in either pure empirics or pure reason”(Bevir, 2010). From pragmatism (Rorty, 1979, 1989), realism (Sayer, 1997; Cruickshank, 2003), poststructuralism (Derrida, 1978), and feminism (Butler, 1992) to conservatism (Oakeshott, 1991), post-Marxism (Laclau, 1989, 1996; Laclau et al., 2000), post-modernism (Lyotard, 1986; Bauman, 2002; Baudrillard, 2007), and analytical philosophy (Quine, 1969; Uebel, 1996; Wittgenstein, 2009; Peters, 2020), all together have aimed to tackle this issue by introducing alternative and contrasting positions. In non-foundationalism, the “bottomless” abyss operates as the figure for a ground that is absent, and no anchoring point can be placed or secured. In this approach, society has to be assumed as an indeterminate and never-ending process with neither an obvious emerging point nor a defined end or destination. Since the abyss is grounded on nothing, its ground is itself (Oakeshott, 1991; p. 60; Marchart, 2007; p. 3). But what we actually experience are sediments and structures, which, as a result of human practices, have been shaped over time through a variety of traditions, cultures, institutions, norms, and so on. These structures have a dual role: on the one hand, they restrict the practices of individuals, and on the other hand, it is in relation to them that the voluntary of individuals finds its implication. Criticism of the foundationalism's premises is essentially the primary concern of the anti-foundationalist approach. According to Marchart (2007) denying the existence of any foundation in the form of a simple reversal of foundationalism causes anti-foundationalism to fall into the trap of foundationalism. There is a necessity to claiming that there is no foundation, a necessity that itself requires a kind of foundationalism. Here, a kind of dualism dominates the anti/non-foundationalist/foundationalist predicament, a dichotomy between “an ultimate foundation and none at all,” (Marchart, 2007; p. 13) or what Fairlamb (1994) considers as “the one-or-none thesis.”

Any encounter with this predicament has to come to terms with the position which goes beyond the mere pluralization of foundations—such as Taylor (1992) and Habermas (2001), who again fall into foundationalism—or absolute denial of any foundations—such as many trends of existentialism, post-colonialism, and post-modernism which turn to the nihilism of absolute abyss. What Marchart (2007) proposes to deal with this issue is “post-Foundationalism.” According to him, rather than launching a direct attack on “foundationalism” or “metaphysics,” what should be done is a subversion of the ground on which foundationalism operates, a subversion of foundationalist premises, instead of their rejection. Post-foundationalism is a kind of deconstruction of foundationalism, what Marchart considers as “a constant interrogation of metaphysical figures of foundation,” (Marchart, 2007; p. 2) or Judith Butler takes as ontologically diminishing the status of foundations without completely eliminating them (Butler, 1992; Butler et al., 2000) and what Spivak (1993) depicts as a persistent concentration on the formation of foundations that are assumed to be “self-evident.” In this way, it is not the existence of the foundations but their ontological status that becomes problematic, a situation that averts social analysis from paying attention to the really existing foundations to their status, that is, their conditions of possibility. Thus, as Marchart indicates:

*The ontological weakening of ground does not lead to the assumption of the total absence of all grounds, but rather to the assumption of the impossibility of a final ground (groundlessness), which is something completely different as it implies an increased awareness of, on the one hand, contingency and, on the other, … [the conditions of (im) possibility of a foundation] … as the moment of partial and always, in the last instance, unsuccessful grounding. (Marchart, 2007; p. 2)*

The claim that a final ground is impossible is necessarily true for all possible foundations. It refers to “the necessary absence of an ultimate ground.” It is important to stress that this is a constructive absence rather than an ultimate negation. The “absent ground” is not an “anti/non-ground” in any sense. The fact that the ground stays present in its absence emphasizes the fact that the ground's absence does not compromise the grounding process would end. Conversely, as Marchart put it, “to some extent the ground remains ‘operative' as ground only on the basis of its very absence, which is why the absence of the ground must not be envisaged as ‘total' cancellation, as ‘mere' absence” (Marchart, 2007; p. 18). Drawing on the Heideggerian point of view, he strives to indicate that the ground is embodied within the abyss and abyss attached to the ground, i.e., “a ground without ground, a bottomless ground” (Marchart, 2007; Heidegger, 2012). As a result, grounding continues to take place. The ground's function as the ground does not fully vanish. Nevertheless, it only happens if it goes through an “abyss,” which is the ground, and means that the foundation grounds as an abyss. Therefore, from a Heideggerian approach, “the abyss remains active and present in the ground as the process of ‘essencing' or holding sway” (Marchart, 2007; p. 18–19; Heidegger, 2012).

In post-foundationalism, propositions such as the presentation of ground in its absence as well as “the necessary absence of an ultimate ground” lead to the priority of “the possibility of contingent foundations in the plural” and also of the position in which “the process of grounding as presencing/absencing dominates the idea of solid and ultimate ground” (procedural groundings) (Butler et al., 2000; Marchart, 2007; p. 15–18, 25). Just as the contingency associated with “contingent grounds” is a necessary contingency, the impossibility of a final, uniform, and present foundation is a necessary condition of possibility for grounds in the plural. The concept of contingency implies that all grounds require a foundation that is neither impossible nor unnecessary (Vallicella, 2002; Trogdon, 2013). Contingency, thus, links the possibility of ground to the impossibility of its complete fulfillment, namely non-contingent, which is emphasized as a contradiction between the relation of possibility and impossibility. In this respect, contingency may be employed as a functional phrase to denote the impossibility of the closure or complete ground actualization. Therefore, by prioritizing contingency in post-foundationalism, the conditions of possibility, as well as the conditions of the impossibility of grounds, are taken into account simultaneously (Butler, 1992; Butler et al., 2000; Marchart, 2007).

One of the consequences of this prioritization is the centralization of a kind of historicism. In other words, if facing contingency is always feasible, even if it was not always realized, then the realization of contingency must be contingent on certain conditions and circumstances (Marjanen, 2009; Conrad, 2016). It relies on what could be considered the “historical constellation” and the “moment” of contingency which is realized from the viewpoint and within a spatial and temporal context (Marchart, 2007; p. 25–33). That moment of encountering contingency is considered the ground configuration in the moment of post-foundationalism. This recognition of historicity is designed to demonstrate that always under a specific constellation the emergence of the post-foundationalist moment would be possible, which itself points out to the actualization of the necessity of contingency and the impossibility of an ultimate foundation. The constellation itself is deeply historical and empirical, pointing to the realization of contingency as a necessity. This necessity is itself an unnecessary consequence of empirical conditions. Just as ontologically, grounds, in the process of (un)grounding, are realized in certain moments and historical constellations, so the significance of contingency and groundlessness is possible epistemologically in a certain historical horizon, that is, the horizon of post-foundationalism.

With the collapse of the markers of certainty and the recognition of the impossibility of a final ground, it will be impossible to find a definitive and solid foundation as the positive ground for the social. In reality, what exists is the plural, particular, and ultimately, failed endeavors at grounding society. The social is avoided from closure, completion, and from becoming identical to itself. From the viewpoint of the impossibility of society (Laclau, 1991), which is the same as the impossibility of postulating a final definition of the social, the moment of complete realization of the social is continuously suspended and what is achieved is always partial. As a result, the concept of foundation implies two related dimensions: a rigorously negative foundation (the impossibility of a final ground) on the one side, and the possibility of ‘contingent foundations' on the other (Marchart, 2007; p. 7). In this respect, every ground would be a partial, uncompleted ground within a field of contesting for foundational efforts. Consequently, society would constantly be on the lookout for a final ground, whereas the best that could be accomplished will be a transient and “contingent grounding,” the plurality of partial grounds which are articulating through a variety of social configurations. But these foundations or configurations will never be able fully to live up to their function as the ultimate ground and configuration. Thus, the configurations are the moment of ground, the moment of the realization of grounds, which will never halt and be completed. In this way, all dimensions of society would be subjected to the continuous play of grounding/ungrounding, a process that manifests itself through partial grounds and uncompleted social configurations. The conditions of grounding/ungrounding of all social beings will determine the quality and quantity of social configurations, this is the very moment of post-foundationalism. Preceding contingency and groundlessness in the inquiry of social configurations reveals that these configurations are actualized in a particular historical constellation and at a specific moment, and are meaningful with respect to that constellation and moment. The moment of ground and social configuration, in turn, is only realizable within a certain historical constellation.

What would be the translation and implications of the post-foundationalist approach to social inquiry? This attempt by society to construct a foundation and to ground the social on an evolving and partial foundation, alongside incessant configurations, on the one hand, pave the way to tackling antinomies such as agency/structure as well as universalism/singularism, and on the other hand could offer a different trajectory to deal with the predicament of essentialism/relativism in social theory. Instead of the social and other definite and distinct objects such as class, nation-state, civilization, cosmopolitanism, or reifying general terms such as culture, religion, politics, modernity, and globalization, rather than taking into account imaginary well-bounded scales such as national, regional, global, and civilizational spaces—abstract categories that are mostly depicted intellectually—from the post-foundationalist approach, and by considering incompleteness, groundlessness, and contingency, and the fact that each foundation comprises the exclusion of other possibilities, social theory could begin its inquiry by examining the condition of (im-)possibility of social configurations at the moment and space of their actualization. But what are the coordinates of these configurations and how are they (de-)constructed? How could we make sense of and conceptualize the configurations based on their special characteristics in relation to their conditions of possibility?

## 3. Social (con-)figurations

One of the manifestations of foundationalism in classical sociological theory has been the emergence of the antinomy of agency/structure or, with a slight difference, the individual/society; In methodological individualism, the agency (and it is voluntary) is considered the solid foundation of the social, and in structuralism, the structures are the ground for the formation and understanding of society and the social. Numerous solutions and alternatives have been proposed to overcome this antinomy (Giddens, 1976, 1984; Habermas, 1984; Bourdieu, 1989, 1990a,b; Archer, 2000, 2003; Sayer, 2010), solutions that, to some extent, have fallen into a kind of foundationalism or a kind of anti-foundationalism. The notion of “figuration” is one of the brilliant suggested ideas to dissolve this antinomy that is proposed by Norbert Elias (1984). By considering the process of construction, reconstruction, transformation, and deconstruction of social figurations, he sought to prioritize interdependency, infinity, procedural, and relationality over determinacy, stability, and independence in inquiring about social phenomena. Elias endorses that the dominance of classical ontology in sociology has led to the supremacy of a kind of essentialism and teleology, an ontological state which has discarded the relationality and incessant nature of social phenomena and considered them as separate, self-found, and reposing entities. This type of social imagination has given rise to some dilemmas as well as antinomies in social explanation such as the antimony of the individual and society, an antimony that has arisen as a result of the perception of the individual and society as two separate and isolated entities based on the two distinct, solid grounds. By considering the contingency and prioritization of the condition of possibility of social phenomena, the idea of social configurations could effectively delineate the interdependency of social actors and their constant transformation in a post-foundationalist approach.

For Elias, figuration signifies the “network of interdependences formed among human beings and binds them together, … [that is to say], a structure of mutually oriented and dependent people” (Elias, 1994; p. 213–14). He considers the individual to be an open and indeterminate being, which is basically oriented toward other persons, almost dependent upon and in need of others with whom she or he constructs complex figurations. As a result of the process of objectification and reification of social phenomena as well as the expansion and heightening of social differentiation, individuals forget their interdependence and openness and assume that social entities “like nations, races or classes have an existence prior to and independently of all of them” (Elias, 2001; p. 85). For him “the concept of individual refers to interdependent people in the singular, and the concept of society to interdependent people in the plural” (Elias, 1984; p. 125) and he considers society as well as social formations as a figuration constructed by numerous interdependent individuals,

*For the fact that people do not exist as isolated, hermetically closed individuals, but as mutually interdependent individuals who form figurations of the most diverse kinds with each other, can be observed and demonstrated by particular studies. In such studies the genesis and evolution of specific figurations can be determined with a high degree of certainty even though they are, of course, only a step on the way. One can ascertain the conditions under which people were mutually dependent in this specific way, and how these dependences evolved in their turn in conjunction with partly endogenous and partly exogenous changes in the total figuration. (Elias, 1983; p. 209)*

In this respect, the central question of sociology is “how and why people are bound together to form specific dynamic figurations” (Elias, 1983; p. 208), a question that can only be answered in terms of the interdependence of people. By scrutinizing how people are connected and reciprocally dependent at different junctures of social development, by striving to illuminate the reasons why the dynamism of human dependence takes on this specific shape and pattern at this point, a better understanding of the formation and evolution of figurations will be achieved.

The emergence, (re)construction, pattern, transformation, and destruction of these figurations do not follow any universal rules. In Elias' conception, a figuration comes into existence when two or more individuals or groups develop some type of relationship that is fueled by their mutual dependency and allows them to exercise reciprocal restriction (Elias, 1984). Figurations comprise varying degrees of stability, harmony, homogeneity, complexity, structurality, regulation, and durability. They are constructed in diverse patterns and coordinates at different times and spaces. The superiority of agency or structure becomes relevant with respect to a specific figuration. In a certain figuration, at a specific moment, the agency of the individual may be decisive, but at other times and situations, the structure plays a pivotal role. On the other hand, this superiority is completely uneven and heterogeneous. Social figurations can be deeply structured and construct an institution, or they can be a momentary construction without a definite and clear boundary. Norbert Elias employs the metaphor of a game (Elias, 1984) as well as a court (Elias, 1983) to describe many figurations. Games are a good example of the interdependence of individuals. Games have different forms and organizations that are performed by different individuals and groups. The actors of these games occupy interrelated positions. Occupying these positions requires learning and practicing some defined roles. The game is somewhat independent of individual players, and encompasses rules that are external and coercive to individuals, and specific to that game, while this game itself is not independent or external to them, that is, the game cannot exist without its actors. In the course of the game, the actions of the actors are adopted in relation to other actors (teammates or rivals) as well as the rules and general flow of the game. While showing some degree of regularity and organization, games are also highly indeterminate and variable. The same rationale can be applied to the emergence, construction, transformation, and deconstruction of figurations. Elias argues that

… *the figuration formed by the players is as concrete as the players themselves. By figuration we mean the changing pattern created by the players as a whole -not only by their intellects but by their whole selves, the totality of their dealings in their relationships with each other. It can be seen that this figuration forms a flexible lattice-work of tensions. The interdependence of the players, which is a prerequisite of their forming a figuration, may be an interdependence of allies or of opponents. (Elias, 1984; p. 130)*

Similar to those in a game, the dynamics formed and shaped in a figuration provide opportunities for it to develop within predetermined patterns, control the risks and impact of threatening factors, and in terms of the outcome of the game, could reduce the uncertainty. But figurations are always exposed to uncontrolled dynamics and factors, elements that threaten their existence. These factors and dynamics either arise from within these figurations or from outside and may either alter or reconstruct them or completely destroy them. Here, the actors' roles as well as their relations change, and the constructive intention around which the figuration was formed is metamorphosed in general. Emergence, consolidation, transformation, and deconstruction of states, families, religions, cities, economic and political systems, and so forth, can be sensible through this kind of post-foundationalist explanation. As mentioned earlier, interdependence and relations are the cornerstones of the formation of figurations. The sharp and sudden rise of actors and the consequent growth of relationships and the deepening of dependencies themselves play a central role in the fluidity, emergence, and collapse of figurations. This is well seen in the process of globalization and the widespread interdependence between groups on a global scale (Luhmann, 2012). As a result of this process, many figurations have collapsed and many more have emerged with new features. This implies contingency and indeterminacy for many of these figurations. In this context, Elias emphasizes that

*It is as if first thousands, then millions, then more and more millions walked through this world, their hands and feet chained together by invisible ties. No one in charge. No one standing outside. Some want to go this, others the other way. They fall upon each other and, vanquishing or defeated, still remain chained to each other. No one can regulate the movements of the whole unless a great part of them is able to understand, to see, as it were from the outside, the whole patterns they form together.... Thus, what is formed of nothing but human beings act upon each other, and is experienced by many as an alien external force not unlike the forces of nature (Elias, 1956; p. 232)*.

The employment of the metaphor of a game and prioritizing the power relations by Elias to describe figurations poses some restrictions on the efficient understanding of social figurations. Although each game has its own rules, Elias sought to tease out universal rules for society from the general metaphor of the game. The question raised here is that in the globalized world, are we facing adult games with higher universalization and consistency or children's games with lower regulation and universalization? New (con-)figurations, exactly like children's games, display different relationships and determinations at the moment of their grounding, even in the same conditions. On the other side, according to Elias, figurations, like games, are formed around meeting a need (the condition for the possibility of forming games as well as figurations). To meet this need, individuals have different accesses and monopolies to resources, which leads to social conflict, competition, and inequality. In this way, notions such as function, power, coercion, unequal relations, subordination, and superordination become central concepts for understanding social figures. Elias, like Pierre Bourdieu, seeks to establish general principles for the study of uneven and ongoing social phenomena in an unessential way. Although this kind of interpretation works for some of the most important figurations, it disregards many other figurations. On the other hand, this conception highlights some features of figurations and ignores others. Here, too, there is a danger of falling into foundationalism in understanding social figurations, because a universal and ultimate ground is perceived for social phenomena. Various types of figurations, along with their fluidity, prevent them from being categorized into fixed and universal types. On another level, Elias' emphasis on power and power balance in the formation or transformation of a figuration ignores the representational, subjective, and cultural dimensions of figurations at a high level (Featherstone, 1987). Thus, recognizing social figurations in the current global situation requires a more indeterminate and heterogeneous understanding of figurations, an interpretation that can make sense of the incompleteness, groundlessness, interdependence, unevenness, and uniqueness along with the subjectivity and positionality of these phenomena as well as their simultaneous regularity, organization, and partial ground. In this context, Rehbein (2015, 2018), by introducing the notion of “Kaleidoscopic Dialectic” in the formation of heterogeneous configurations, strives to present a more indeterminate interpretation of the idea of configuration, and thus eliminate another antinomy in social thought, that is, the antinomy of singularism and universalism.

An intricate and fundamental predicament in understanding social phenomena is whether they embrace universal rules, that is, whether it is possible to consider solid foundations for them, or whether they are singular and specific phenomena and events, and have no universal rules, that is, whether they are principally groundless. By assuming regulated societies and social objects, sociology, since its inception from Auguste Comte to many of its later versions, has sought to derive different laws and causal chains with different generalizability and universality. As noted earlier, the very idea of there existing an independent phenomenon called society, which has sound foundations and is self-ruled, is a step into the world of foundationalism. On the other hand, many criticisms of this approach have provided the basis for a kind of extremist ungrounding in the form of singularism. By radicalizing the idea of Elias' figuration, the idea of social heterogeneous configurations could propose a more efficient conceptual apparatus for understanding social phenomena in uncertain and indeterminate global terrain. For Elias, “figurations” are specific formations of human beings that have been constructed in interconnected relationships and in a conscious and intentional way, while “configurations” are specific to non-human, abstract, and physical phenomena (Elias, 2003). But, inspired by Theodore Adorno's notion of the constellation, Boike Rehbein attempts to promote the idea of figuration by using the concept of configuration. Rehbein aims to offer a mode of social interpretation appropriate to the current post-colonial, post-Eurocentric, multicentric, pluralistic, and interdependent world. He argues that his solution is going beyond the predicament of relativism and essentialist universalism, a predicament that is itself another manifestation of the problem of foundationalism and anti/non-foundationalism. Drawing on Gadamer (2004), he states that knowledge of the society is possible only within the framework of existing society, based on its own history, and by the means it produces (Rehbein, 2018; p. 57). Nevertheless, in the post-Eurocentric world, this situation must be re-interpreted in a completely new way, because the history that contemporary social theory must scrutinize is no longer a uniform, universal history with a uniform, single European-centered ground. Based on his propositions (Rehbein, 2015; p. 13, Rehbein, 2018; p. 52, 57) it can be claimed that, on the one hand, there are no conditions of possibility for transcending the existing society—and its history—and finding a fixed ground outside it, and on the other hand, in the current world, we are encountering a variety of distinct and “non-identical” foundations.

Based on general propositions or universal “laws,” and because of the endless relations and causal chains, according to Rehbein, there are many possibilities to explain any hypothetical phenomenon. “Each level of explanation, each interest, each discipline, each method, and virtually each glance results in a different description of the phenomenon, even if it remains identical” (Rehbein, 2015; p. 34, Rehbein, 2018; p. 59–60). This leads to pluralism in foundations. Consequently, we are in a dilemma between the arbitrary reduction of unlimited foundations to a limited and universal foundation or law, or mere acceptance and submission to the abyss. Therefore, he argues for a position that considers the multiplicity and incompleteness of the foundations; and in taking into account social phenomena, it avoids the universal laws, totality, and the idea of the unilinear evolution of history (toward an ultimate goal). He urges an epistemological stand that is neither purely descriptive nor universalizing and deductive (Rehbein, 2018; p. 57). Rehbein argues “kaleidoscopic dialectics” to be the best way to overcome these predicaments (Rehbein, 2015, 2018).

For Rehbein, this approach, as a relational method, aims to scrutinize heterogeneous configurations, the formation of their constructive relations, and their history. Relations here also signify a variety of relations from contradictions, temporal succession, competition, similarity, and attraction, to generation, domination, consensus, and camaraderie. Kaleidoscopic dialectics entails three fundamental qualities. First, the object of inquiry must not be construed as a configuration at the level of the particular but as something between the universal and the singular. Second, configurations must be attributed to the clearly defined empirical realm. Third, the configurations must be constructed historically, but without any teleology or mention of an origin or destination (Rehbein, 2015; p. 90–100, Rehbein, 2018; p. 58). Abstract interpretations of history and social objects make them seem universally applicable. But in reality, a law will be applicable only within a particular realm of phenomena with which it has emerged at a historical moment. This is the very meaning of the ‘particular' for Rehbein. “Some laws and rules apply to many phenomena, some too few—however none to all and none to just one” (Rehbein, 2018; p. 60). Thus, each configuration implicitly refers to universal propositions and laws, but they apply and are true only to the relevant configuration. Each configuration remains open and endless as new relations emerge and new relations are uncovered. Drawing on Wittgenstein's idea of family resemblance (Wittgenstein, 2009), Rehbein attempts to reveal that configurations cannot be reduced to universal similarities and general concepts because these similarities and concepts do not suffice to fully grasp them (Rehbein, 2015; p. 97, Rehbein, 2018; p. 61). It means that they have similarities in some relations and aspects, but at the same time, many different in the others.

Various types of relations between social configurations could be explained by tracing their history—by taking into account their ruptures and continuity—while they cannot be reduced to universal laws and history. Hence, social configurations do not end at the moment of their crystallization as well as their explanation, and will always be indeterminate and endless in terms of their internal and external relations. According to Rehbein, one can always discover new relations and family resemblances, which means that there is no ultimate and universal explanation for the configurations. The only thing that can be declared here is that there are configurations that are more universal than others because they include more objects, categories, individuals, groups, and relations. Social inquiry, therefore, must look for configurations at the particular that are empirically saturated and clearly defined in scope, and do so by discovering and analyzing as many relations as possible. What is going on in the real world is the confrontation and synchronicity of heterogeneous, self-reflexive, and incommensurable configurations (Rehbein, 2015; p. 104). They have their own particular boundaries and plausibility. Since the social configurations and the corresponding cognitive configurations are increasingly intertwined and colliding with each other, they can no longer be considered in isolation as distinct entities. There is no fixed, universal foundation that can explain the types of configurations and their relations. A kaleidoscopic dialectic, as Rehbein (2018; p. 62) put it, investigates which configurations could be applied to which realm of social objects by confronting them with each other without assuming a general explanation and universal law as well as a fixed and ultimate ground.

By revisiting Elias' idea of figuration and drawing on Foucault's idea of dispositif and Adorno's notion of the constellation, Boike Rehbein strives to propose the idea of kaleidoscope dialectics and the centrality of configurations. But these philosophical foundations and premises at a high level depart from the central idea of Elias in determining (con-)figurations as units of analysis. As noted, Elias' idea of figuration, finally slips into the trap of foundationalism, but by overcoming some restrictions of the figuration theory, Rehbein's idea of heterogeneous configuration and his emphasis on the relational aspects of the social objects can be promising for social analysis in the era of cosmopolitanization. It can be argued that Rehbein's contribution remains mostly at the theoretical level. What he formulates are epistemological suggestions for correcting and relocating critical theory in a multipolar world–a world about which, ontologically, he has no clear idea and therefore cannot attribute his epistemological apparatus to it. His idea of empirical investigation has no practical tools to grasp the social and hybrid configurations in their ongoing process. The particular and the particular level do not have a specific empirical meaning and determination. The structural context and conditions for their construction are largely disregarded, and a specific idea about space and time, as well as a theory of action, are absent from his conception of configurations. But we need an idea of configuration that, while suspending the dominant theoretical categories in social inquiry, posteriority, and empirically, is able to conceptualize and make sense of configuration's features, such as its categories, orders of category, relations, etc., within a certain spatial and historical constellation and with respect to the moments of its actualization. In some respects, configurations are much more indeterminate, and in other aspects, times, and places, they are much more determinate than what Rehbein is striving to prove. Therefore, any social analysis should begin by addressing social actors at different social levels. But Rehbein's conception of social configurations is largely trapped in the trap of reproductionism, which is also well seen in Bourdieu. His interpretative method is regressive and oriented toward the past, which is a non-historicist approach. The idea of constructing social realities, which can be extracted from a special reading of Bourdieu's theory of action, compensates for the shortcomings to a large extent.

## 4. Formation of social configurations: Categories construction, boundaries making, and orders of category

By suspending the conventional concepts, categories, and methodologies in the social sciences, consequently, any social inquiry in the current fluid world, must begin its work by examining the quiddity of social configurations in the moment of their actualization, especially with respect to their constitutive historical constellation. Centering on the empirical context and categories in practice, as well as prioritizing the conditions of their possibility, are the starting point for the analysis of these fluid, uneven, and indeterminate entities. Individuals with expected intentions and aims that are themselves fluid and indeterminate and are highly subject to these configurations, build different types of social relations and come to interact with each other. The result of these relations is the construction of various social configurations with certain boundaries, rationality, structuring, power relations and inequality, cultural compromise, conflict, differentiation, groupness, identification, systems of meaning-making, etc. Drawing on the post-foundationalist and relational approach, it is critical to consider that all these categories, ingredients, and items are all constructed a posteriori within the framework of social configurations and can be understood and interpreted based on them. In this case, many methodological problems in social theory, including the problem of essentialism and relativism can be solved. Since these social items are relationally and procedurally constructed through an uneven network of relations (Mohr, 2013) in a certain historical constellation, it is no longer possible to speak of fixed, isolated, and eternal categories such as nation, state, class, and identity, categories for which their determinants are uneven, fluid and multifaceted. The nature of the categories and their level of determinability depend on the coordinate of a particular configuration, namely a heterogeneous configuration that immediately prioritizes other categories and relations at another level. Thus, according to Norbert Elias, we are confronted with open human beings who are the creators of the configurations and their elements that are relationally, interdependently, and procedurally, constructed. Therefore, instead of identity, for instance, we should talk about identification (Brubaker, 2004). Essentially, the problematization of identity itself is relative and dependent on the coordinate of a specific configuration at a particular time. Also, instead of groups and groupings with rigid boundaries, we should talk about groupness; this means that we are faced with an uneven range of social differentiation (Brubaker, 2004; p. 7–28). These configurations are mainly formed around a specific order of categories, a fluid order that determines many of the characteristics of these configurations, including their boundaries, structuring, and universality.

By deferring abstract, imaginative, and detached concepts such as society and the social, according to Pierre Bourdieu,

*The object of social science is a reality that encompasses all the individual and collective struggles aimed at conserving or transforming reality, in particular those that seek to impose the legitimate definition of reality, whose specifically symbolic efficacy can help to conserve or subvert the established order, that is to say, reality. (Bourdieu, 1990b; p. 141)*

Here, social reality can be considered a set of heterogeneous social configurations, which in different orders and relations to each other, and different historical constellations, have been the source of various kinds of social manifestations. In this approach, all social manifestations are the outcome of the efforts of society and social actors to build a solid ground for social order and its relevant meanings and categories. These configurations and collective manifestations are contingent possibilities among many other contingent possibilities that have been realized under certain conditions of possibility. Thus, social actors can be envisaged in an open, endless, and unstable process in social negotiation, in which actors must possess some preconditions or a priori categories to enter the negotiation process. During this negotiation, some new relations, categories, and common symbolic patterns are generated. These common categories and symbolic patterns become the basis for some social practices and varying degrees of social demarcation, discrimination, and differentiation (Foroutan, 2018). In order to be able to take part in a social negotiation and promote their social practices, in a Bourdieusian approach, it can be said that individuals have to internalize their positions in social terrain by steadily developing a habitus fitted to these positions (Bourdieu, 1990b; Jenkins, 1992; Todd, 2005). The habitus is a system of “predispositions” that establish practices, perceptions, and interpretations regarding related social positions (Bourdieu, 1990b; Bourdieu and Wacquant, 1992). The outcome of this process is the construction of a collection of strategies for action and cognitive patterns. In this respect, Andreas Wimmer considers “Scheme” as the empirical representation of habitus. “Schemes are models of simplified worlds,” he argues, “ordered as interconnected networks of meaning [and relation] and they are spontaneously selected and activated in everyday perception, consciousness, thinking and action” (Wimmer, 2002; p. 27). Individuals internalized these schemes of cognition within their own lifeworld through socialization processes and everyday social interaction. In other words, habitus could be considered as “being formed based on […] the competence of assessing pros and cons in given situations in light of one's own interests” (Wimmer, 2002; p. 27–28). The perception and evaluation of “what one's own interests are” relies on initial regulations to social and cultural backdrops and one's own social position in a social configuration.

The entry of individuals into the process of social negotiation and their interaction with each other provides the basis for building new types of relations and new social categories. In this regard, Wimmer maintains that when virtually “habitual schemes” are customized to the distinct positions within a society, they construct different categorizations, classifications, evaluations, and world-views. On this ground, they produce categories and symbolic nodal points with different orders which all actors who are involved can identify as “congruent” to their corresponding interests (Bourdieu, 1990b; Wimmer, 2002). The negotiation process eventually provides the conditions for the possibility of different configurations. At this level, a key element is the crystallization of different types of “cultural constructions or compromise.” Considering the notions of negotiation and consent, Wimmer defines cultural compromise “as consensus over the validity of collective norms, values, categorizations and patterns of interpretation that persists beyond the open and endless process of its construction” (Wimmer, 2002; p. 29). In everyday life and interaction, the actors negotiate: how a position ought to be defined, who should play which kind of role, which plans for action should be pursued, and which norms and values are relevant in the certain position.

Thus, when people assent around some interests, then any binding rules for collective making-meaning will develop. Therefore, a social configuration will appear when all social actors, based on their interests, in relation to each other in a commutative field, come to a consensus around social categories and classifications, and then they are trying to make them legitimate and valid (Brubaker, 2004). The outcome of this process would be different types and orders of social categories which represent themselves through various national, ethnic, racial, etc., order of categories (Wimmer, 2002; p. 28–33). This moment can be considered as the temporary moment of actualization of social foundations, a point that is determined as a result of a certain historical constellation. These categories are formed temporarily and uncompleted in relation to other (existing) elements, and their significance can be grasped in relation to the new configuration in which they have emerged. In the new configuration, hence, many of the existing categories and relations will find new implications. The order of these categories, their arrangement, the types of their syntagmatic and paradigmatic relations, and, in general, their grammar at a high level, determine the coordinates and quiddity of the configurations. The grammar of these orders of category and the dependent regime of boundaries disclose the types of national, transnational, religious, cultural, political, etc., orders of category in a specific configuration. The precedence and determinism of the culture in a certain configuration, for instance, can be proved on the basis of a particular order of category, at a specific moment. One can speak of the economic nature of a phenomenon when the order of categories in a particular configuration is such that economic relations dominate and have decisive power. Consequently, we have nothing economic, cultural, or hybrid in essence. In this way, many theories on hybridity or intersectionality, theories that continue to presuppose separate entities, become irrelevant. In a configuration, one may identify oneself (or another person) by one's position in a relational network (for example a network of kinship or friendship, patron-client ties, or teacher-student relations) or one may identify oneself (or another person) by membership in a class of persons sharing some categorical attribute (such as race, ethnicity, language, nationality, citizenship, gender, and sexual orientation) (Brubaker, 2004). It is an endless chain of evaluation and identification that has been temporarily halted in a configuration.

The construction of many of these categories, relations, and elements pave the way for the formation of various types of social exclusion, closure, boundaries, and demarcations that themselves represent the scale and scope of a configuration on an empirical level (Lamont, 2000; Lamont and Molnár, 2002; Lamont et al., 2007). By drawing a dividing line between the familiar and the foreign, insider and outsider, through the process of social closure, those who are not on the same side in terms of sense of belonging, categories, identity, etc., will be excluded. In relation to the order of categories, the clarity and permeability of the boundaries, and the degree of structuring of the configurations, social closure could result in the formation of different social differentiation, groupness, and entitles like classes, gender-defined groups, subcultures, or ethnic groups, nations and transnational diasporas, fields, domains and so forth. The boundaries between “us” and “them” are frequently marked by distinguishing shapes of everyday practices (Wimmer, 2002; p. 33-34), which vary the configuration bounds in a relational way (Mohr, 2013). So, it can be said that such cultural compromise and the order of the categories are constructed if all social actors in relation to each other in a social configuration can formulate their interests in “a shared symbolic language and categories.” In conclusion, an outcome of this negotiation would be “certain social markers”, i.e., configuration, which is singled out in order to expose and support the distinction between insiders and outsiders—between those who are in the same configuration and those who stay on the margins.

In this respect, several parameters play significant roles in constructing social configurations in a given social context in a certain time and space. Individuals or groups who are participating in the process of negotiation, types of involving interests, the given setting, types and orders of symbolic and material resources as well as the level of accessibility to them according to different groups, different encounters with the categories and their subjective interpretations, different modes and regimes of boundaries, regimes of power, etc., can all together be considered as the conditions of possibility and transformation of social configurations. These configurations are in a constant state of change according to their internal and external relations with other configurations, as well as their conditions of possibility (Mosleh and Jong, 2021). In order to avoid falling into the trap of dominant antinomies and predicaments in the social sciences, antinomies and biases such as foundationalism/non(anti-)foundationalism, agency/structure, the individual/society, essentialism/relativism, universalism/singularism, and by considering the contingency of social phenomena and prioritizing the condition of their possibility, any social inquiry, in the highly complex and intertwined world, must begin its work by analyzing these heterogeneous social configurations and their main features.

## 5. Conclusion

Modern social sciences were formed in a moment of classical modernity in which the human world was imagined as if it had universal laws and standards, exactly like the natural world. Scientific knowledge is perceived as recognizing these laws and proceeding on the basis of a kind of universal, formal, and predictable rationality. The outcome of this course has been the formation, reconfiguration, and consolidation of various social institutions in the modern world. The salient feature of this classical modernity, first modernity (Giddens, 1990; Beck, 1992) or solid modernity (Bauman, 2000b, 2002), in the words of Andreas Reckwitz, was that it systematically sought to gain the total generalization, schematization, standardization, reification, and universalization of all social structures and processes, of subjects and objects, of individuals and groups, and procedures which were associated with the process of formal rationalization (Reckwitz, 2020b). The formal rationalization, in its ontological, epistemological, and normative faces, has preached a kind of social universalization. In this universality, which presupposes a kind of ontological unity between the natural and the social, “all potential elements of the world are observed, evaluated, produced, and adapted as copies or instances of generally valid patterns” (Reckwitz, 2020b; p. 143). In formal rationality, the knowledge of all social objects and the rules that govern them are based on a uniform, solid and universal ground, which is itself the basis for institutionalizing those rules and achieving the highest certainty and efficiency. In social theories and inquiries, objects are generally constructed and exercised in a standardized, regulated, and identical way. Recognizing an object encompasses knowing all similar objects at all times and places.

But the first or classical modernity has given way to the late, liquid, cosmopolitan, and risk modernity in which the transformation of industrial economics, from mass production to cultural production, individualization, and consumer society, alongside the rapid spread of media technologies, all together set the ground for the reconfiguration of society and its relevant elements (Giddens, 1990; Bauman, 2000b; Beck, 2016d; Jong, 2016b; Reckwitz, 2020a). In this world, rather than experiencing a single, uniform, and integrated foundation in rationality, history, institution, state, economy, and culture, we are encountering multiple and diverse configurations whose fluidity, indeterminacy, unevenness, plural relationalities, and procedurality are their hallmarks. In this multi-layered and uneven world, social actors and phenomena are in constant but fluid relations with each other at various levels, fluid interactions that have themselves given rise to various kinds of social configurations at different social scales and ranges. Both ontologically and epistemologically, many existing and dominant entities, boundaries, concepts, notions, categories, and ideas have lost their significance. But this does not mean that established and institutionalized phenomena and entities, such as the state, bureaucracy, economic structures, and cultural traditions have been completely deferred, yet they have acquired new implications and functions. The new implications will be significant in terms of the growing fluidity and interdependency in recent globalization. These transformations, above all, illustrate the necessity of utilizing a post-foundationalist approach to understanding new social phenomena on a global scale.

Given the incompleteness of society and the social, any social analysis must primarily consider the contingency as well as the conditions of possibility of a social phenomenon, i.e., a configuration. This means that one should be aware that the actualization of these configurations, which are themselves a crystallized possibility among a variety of different possibilities, is feasible in certain circumstances. Configurations themselves are determined based on certain orders of category and their associated boundaries and are always subject to profound changes in terms of the relations of these categories as well as contextual conditions. The nature of a configuration can also be understood in terms of its internal coordinates and relations that underlie it. There are no universal rules for understanding them and only some similarities between them can be perceived. Since they are formed at the level of the particular and based on partial grounds, these entities possess varying degrees of institutionalization, durability, universality, and boundaries, and are therefore heterogeneous and incommensurable. This state, in the normative aspect, will lead to the recognition of different kinds of orders of category and non-Western, formal, or modern rationalities. Touching on and recognizing a configuration requires knowing the constituent relations, its order of categories, and its boundaries which will only be possible by understanding the rationality of that particular configuration.

## Data availability statement

The original contributions presented in the study are included in the article/supplementary material, further inquiries can be directed to the corresponding author.

## Author contributions

The author confirms being the sole contributor of this work and has approved it for publication.
